# Origination of the Protein Fold Repertoire from Oily Pluripotent Peptides

**DOI:** 10.3390/proteomes2020154

**Published:** 2014-03-25

**Authors:** Ranjan V. Mannige

**Affiliations:** Molecular Foundry, Lawrence Berkeley National Laboratory, 1 Cyclotron Road, Berkeley, CA 94720, USA; E-Mail: rvmannige@lbl.gov

**Keywords:** pluripotent hypothesis, protein fold invention, oily peptides, origins of life

## Abstract

While the repertoire of protein folds that exists today underlies most of life’s capabilities, our mechanistic picture of protein fold origination is incomplete. This paper discusses a hypothetical mechanism for the emergence of the protein fold repertoire from highly dynamic and collapsed peptides, exemplified by peptides with high oil content or hydrophobicity. These peptides are called pluripotent to emphasize their capacity to evolve into numerous folds transiently available to them. As evidence, the paper will discuss previous simulation work on the superior fold evolvability of oily peptides, trace (“fossil”) evidence within proteomes seen today, and a general relationship between protein dynamism and evolvability. Aside from implications on the origination of protein folds, the hypothesis implies that the vanishing utility of a random peptide in protein origination may be relatively exaggerated, as some random peptides with a certain composition (e.g., oily) may fare better than others. In later sections, the hypothesis is discussed in the context of existing discussions regarding the spontaneous origination of biomolecules.

## 1. Introduction

Proteins are one of the most diverse classes of molecules in the living world, with examples crucial to most life processes [1,2]. Supporting this enormous diversity in function (and dysfunction) is a diverse repertoire of protein folds: specific three-dimensional motifs that globular protein chains assume [3]. How did the versatile repertoire of protein folds come to exist? This question is especially interesting because protein precursors—amino acids and random peptides—existed long before the first functional protein debuted into the living world [4,5,6,7,8,9,10,11]. This is evidenced by the abundance of amino acids in abiotic environments (such as meteors/comets [4,5,7] and pre-biotic Earth [8,9]) and potentially pre-biotic mechanisms for peptide bond formation [10,11,12]. Thus, regardless of the protein-world or RNA-world hypotheses, understanding the transition from random peptide to functional fold may be important in understanding the origin of the earliest successful lifeforms.

Unfortunately, understanding this transition through the observation of historical evidence is impractical, since, despite the continual emergence of new organisms and protein functions, it appears as though the “*ab initio* invention” of new protein folds has not been evidenced since early evolution. This is concluded from the ubiquity of the protein fold repertoire throughout all lifeforms [13] (e.g., the same rhodopsin fold enables both humans and bacteria to sense light [14]) and the inability of convergent evolution [15] and lateral gene transfer [16,17] to facilitate this ubiquity. In the words of Chothia and colleagues [13]:

“The earliest evolution of the protein repertoire must have involved the *ab initio invention* of new proteins. At a very low level, this may still take place. But it is clear that [today] the dominant mechanisms for expansion of the protein repertoire, in biology as we now know it, are gene duplication, divergence, and recombination” (Chothia *et al.*, 2003, Science, vol 300 p 1701; italics added).

“*Ab initio* invention” or the origination of new protein folds appears to have gone extinct soon after the emergence of the first successful lifeforms, after which, the Jacobian form of evolution [18] by incremental “tinkering” (via processes such as recombination, insertion and duplication [13]) rather than *ab initio* “inventing” appears to have become more dominant. Consequently, despite useful discourses [19,20], the development and validation of general theories describing the origination of today’s protein repertoire have been impeded. This paper provides a hypothesis (Box 1) that indicates that some peptides are more evolvable than others [21] and, as evidenced by proteome analysis [22], may have dominantly partaken in *ab initio* fold invention.

**BOX 1****Hypothesis:** Dynamic, degenerate and collapsed peptides (especially oily peptides) are the best substrates for the origination of protein folds.**Evidence** can be found in: (i) traces within proteomes [22]; (ii) lattice model simulations [21]; and (iii) diverse reports (reviewed in [23]) on the utility of dynamism in evolvability [24,25,26,27,28,29,30,31,32].

## 2. Pluripotent Hypothesis

The “pluripotent hypothesis” that this paper proposes is the following: peptides (and proteins) that conform to a multitude of collapsed and interchangeable configurations (thereby being both structurally dynamic and collapsed) will have a higher chance of serving as originators of (potentially many) novel folds. Additionally, oily random peptides are expected to present all these properties and, hence, are expected to have dominantly participated in *ab initio* [13] fold invention.

**Figure 1:**
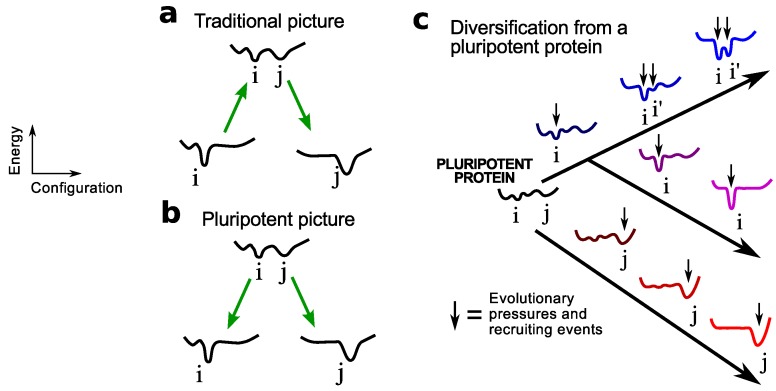
Modes of protein fold invention. This figure depicts the two scenarios for the origination of a new protein fold j described by sequence *j*: (**a**) from a stable protein sequence *i* that describes fold i (arbitrary one-dimensional sequence-dependent configurational free energy landscapes are shown), and (**b**) from a pluripotent peptide sequence *ij* that describes both folds i and j (among possibly others). This paper focuses on the latter mechanism (expanded in **c**), where a collapsed pluripotent peptide may give rise to many folds (i, i’ and j) through miscellaneous recruiting events. As discussed in the text, an expected outcome of this mechanism will be the emergence of today’s proteomes from more oily ancestors. The pluripotent mechanism is qualitatively an extreme version of Jensen’s differentiation model [33] that explained the emergence of protein families and superfamilies [34] as a consequence of differentiation from earlier proteins with ambiguous functionality [33].

These protein/peptide originators are termed pluripotent because, unlike the emergence of novel proteins from a stable protein (Figure 1a), this mechanism of fold invention (Figure 1b) allows for the potential for one peptide/protein to emerge (from, say, replication and mutation) into one of many diverse potential folds (Figure 1c). This mechanism involves an inherent pluripotency, which is a term borrowed from developmental biology that indicates that a pluripotent protein/peptide possesses the potential to be evolved or “differentiated” into one of many folds. In this sense, the pluripotent mechanism is qualitatively an extreme version of Jensen’s differentiation model [33] that explained the emergence of protein families and superfamilies [34] due to their differentiation from earlier proteins with ambiguous functionality [33].

Structural dynamism and the display of multiple transient folds by a pluripotent peptide must play a crucial role in its evolvability (discussed in the context of extant proteins in [23]). Given that folded proteins cannot compete with the dynamic nature of a pluripotent peptide, this hypothesis also indicates that stable proteins are not as fold-evolvable as pluripotent peptides, which are expected to go extinct soon after the first repertoire of folds emerge (see Discussions). This is one explanation for why new folds have not been dominantly emerging in the last couple billion years (since the diversification of the last common ancestor) [13].

As discussed in [21] and below, oily peptides (e.g., peptides that display a high fraction of the four most hydrophobic amino acids on the Kyte-Doolittle scale: F, I, L and V) possess properties such as structural degeneracy, dynamism, and “collapsedness” [21], and therefore may be classified as pluripotent peptides. Given this biochemical link between hypothetical pluripotent peptides and random oily peptides, we next present evidence that oily peptides are associated with fold-evolvability and the development of the first proteomes.

## 3. Evidence

### 3.1. Evidence of an Oily Ancestor within Today’s Genomes

**Figure 2:**
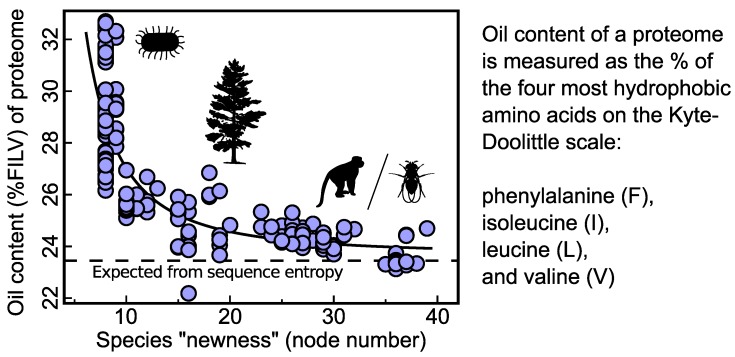
Oil escape indicates a universal ancestor that had an oily proteome. A strong negative correlation exists between a species’ proteome oil content and its extent diverged from the last common ancestor (the node number within the tree of life) [22]. The general trend appears to also exist within distinct types of organisms (e.g., bacteria, metazoa, plants, fungi [22]) and suggests that the last common ancestor displayed a proteome with higher-than-average oil content (which is key for the pluripotent hypothesis discussed here). This inference is not modified by varying metrics of protein oil content [22] and improvements in estimating the tree of life (arising from, e.g., new metrics of speciation for prokaryotes *vs.* eukaryotes, or more accurate estimates of bacterial biodiversity) [22]. Data are adapted from Figure 2 of [22] (silhouettes are provided to convey that a broad range of species were studied).

The pluripotent hypothesis posits that oily pluripotent peptides were important in the origination of the first protein folds. An outcome of this prediction is that the last common ancestor’s proteins would have displayed higher-than-average oil content. This indeed has been evidenced by a recent proteomics study [22] (reviewed in Figure 2). The study reported a universal trend found in all tested proteomes—“oil escape” (Figure 2)—that could not be explained by trends previously reported [35,36,37] and which indicated that the last common ancestor had a proteome with higher-than-average oil content [22]. Oil escape, by indicating an oily last common ancestral proteome, supports the hypothesis that modern protein folds emerged from oily pluripotent peptides.

### 3.2. Lattice Models Indicate Oily Proteins Are Universally Fold-Evolvable

**Figure 3:**
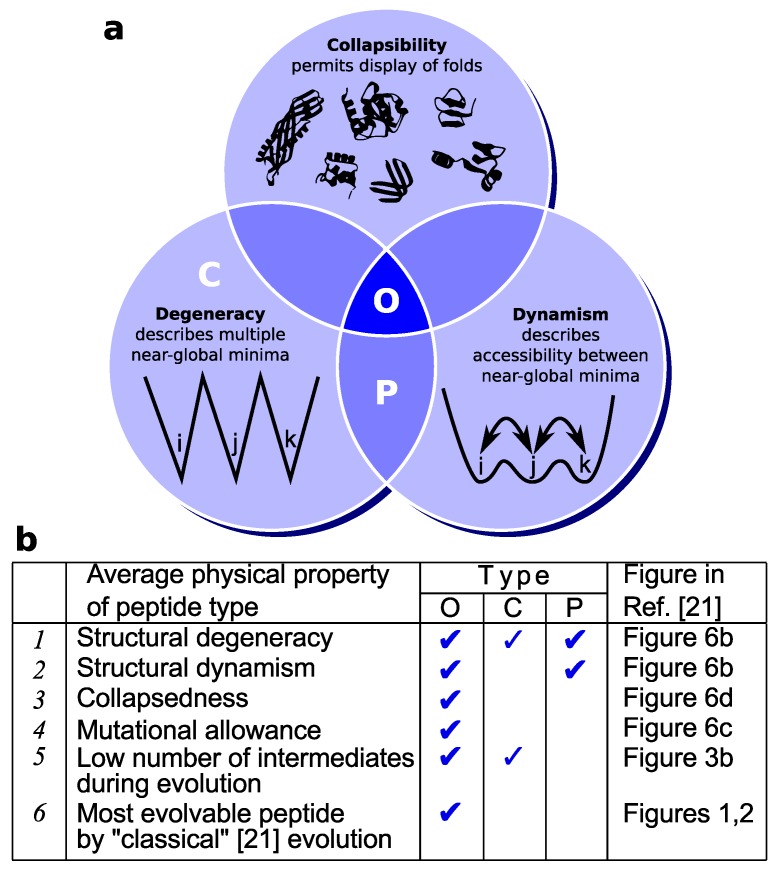
Oily (pluripotent) peptides (“O”) possess many structural and “designability” properties essential to fold invention. (**a**) On average, while most classes of random peptides (e.g., charged “C” and polar “P” peptides) display some of the three properties important to evolution [21], only oily peptides (“O”) describe them all. This may render oily peptides as a superior substrate for protein fold invention. Panel (**b**) relates these structural properties (Points *1*–*3*) to evolvability (Points *4*–*6*). Particularly, directed evolution simulations [21] indicate that oily peptides accommodate the greatest number of non-deleterious mutations (*4*), approach an evolvable target fold through the fewest number of intermediates (*5*) and, due to these features, earn the distinction of being the most evolvable peptide type (among even evolved proteins [21]) for the *ab initio* [13] invention of classical [21] folds (*6*). Key: O, C and P represent peptides with a high percentage of oily, charged and polar amino acids, respectively, defined in [21] by the percentage of the following sets of amino acids, respectively: {F,I,L,V}, {E,R,D,K}, {S,T,N,Q} (single-letter amino acid residues used). Discussions are adapted from [21].

The trace evidence in proteomes, discussed above, is also supported qualitatively by lattice model simulations of proteins [21]. In this study, a positive relationship was found between the oil content of peptides/proteins and their evolvability into target lattice folds. The results indicated that oily peptides are, indeed, a superior substrate for the invention of folds (of “good” design [21]) compared to other random peptides and well-folding proteins (see Figure 3 for a summary).

Mechanistically, oily peptides appear to excel at fold evolution due the following reasons that appear to be a direct outcome of the properties of a pluripotent peptide (Figure 3) [21]: (1) they are more amenable to sequence mutations (they are mutationally “plastic”, due to a shallow and smooth structural free energy landscape [21]); and (2) they readily display either the actual target fold or a related intermediate within the peptide’s native state structural ensemble, on account of having multiple equivalent and accessible near-global structural minima that describe collapsed folds (also, see Discussions on dynamic frustration below). The latter point again brings to mind the notion that these peptides are truly “pluripotent” or capable of evolving efficiently into myriad protein folds (Figure 1c).

The lattice-model evidence discussed above re-asserts the hypothesis that pluripotent peptides (random oily peptides) are the most efficient substrates for the evolution of novel protein folds in a prebiotic setting.

### 3.3. Dynamism in Extant Proteins

The pluripotent hypothesis is strongly dependent on the utility of structural dynamism in the evolution of new protein folds. This notion is an extension of observations from recent protein evolution, where the emergence of a new functionality/structure from a preexisting and stable protein is enabled by a dynamic protein intermediate that describes both new and old structures/functions (Figure 1a) [24,25,26,27,28]. This link between dynamism and evolvability is not new; it was proposed by Landsteiner [30] and Pauling [29] in the 1930s and 1940s and went into dormancy until only recently [25]. Today, Landsteiner and Pauling appear to be corroborated, as the affinity maturation of naive antibodies is concomitant with a transition from more to less dynamic binding sites [31,32,38]. Overall, the discussed link between evolvability and dynamism fits well with the utility of dynamic collapsed structures in the pluripotent hypothesis.

## 4. Discussions

### 4.1. Different Types of Frustration

The principle of minimal frustration has been useful in explaining why an evolved protein often folds reliably to its final native conformation or fold [39,40]. Given that frustration is an unalienable feature of all random heteropolymers [40], one cannot easily minimize this feature in random peptides. However, the display of dynamic frustration, where many metastable conformations are easily accessible (Figure 3a), may be tuned by sequence composition in random peptides (*i.e.*, one can attempt to minimize barriers between metastable conformations). The pluripotent hypothesis posits that dynamic frustration is important to the evolution of a random peptide into any of the metastable conformations available to it. This is particularly important, because all instances of the dynamically frustrated peptide displays many (or every) metastable conformation within its lifetime, which is not the case for a frustrated system that, in each instance of the molecule, gets irreversibly stuck in one of its minima. Dynamic frustration affords the peptide an element of structural dynamism that is known to be important in the emergence of novel protein features (reviewed in [23]). In accordance with this relationship, random oily peptides display high dynamic frustration (compared to random charged peptides) and appear more evolvable to their myriad collapsed states by lattice model simulations [21].

### 4.2. Aggregation

It is important to recognize that, while the capacity for oily peptides to aggregate is high, this property does not preclude their utility in prebiotic protein origination for the following reasons:(i) Aggregation, which is potentially detrimental in biological situations [41], is not shown to be detrimental to protein invention in prebiotic situations; (ii) Aggregation is primarily a concentration-dependent phenomenon made more relevant in closed (cellular) environments [42]; in absence of high local concentrations within putative prebiotic settings [10], the option of aggregating with molecular partners may be less open to an oily peptide than the option of self-collapsing (which is always present in the right environment); (iii) Upon the increase in local concentrations, aggregation, even if prevalent, may actually be useful in bringing molecules together to perform novel, biologically important functionality [41,43,44,45].

### 4.3. Expected Extinction of Pluripotent Peptides and Cessation of Mass Invention

When made to co-exist with the eventual development of stable proteomes, pluripotent peptides would be rendered extinct relatively quickly in a biological setting. This is expected to be the case for two reasons: (i) as described above, aggregation described by oily peptides is potentially detrimental in confined areas at high concentrations; and (ii) in a competitive closed (cellular) environment with limited resources, pluripotent peptides, with no other activity other than being a nursery of as yet nonexistent functionality, would be out-competed for resources by the battery of well-functioning proteins, eventually leaving our last common ancestor’s proteome with few or no vestigial pluripotent peptides. The final outcome would be the veritable extinction of the pluripotent peptide species, followed by the cessation of novel fold invention. This aspect of the pluripotent hypothesis is one explanation as to why significant protein fold invention in “recent” years (the last couple billion years) has not been dominantly witnessed [13].

### 4.4. Intrinsically Disordered Proteins Today

Intrinsically disordered proteins (IDPs) are a highly dynamic class of proteins that are degenerate in their native state ensembles, which confers upon them the capacity to bind to a diverse range of molecular partners [46]. While these proteins are highly dynamic, they lack one major structural element for fold invention that the current hypothesis requires: the presence of multiple collapsed forms (proto-folds) within the native state ensemble (Figure 3). Furthermore, in non-protected environments, an IDP’sextended backbone would be more chemically degradable [19] and, therefore, less capable of sticking around for (pre)evolutionary recruitment of protein folds. This is reflected by the observation that the incidence of IDPs within the proteomes are low in prokaryotes and increase within complex organisms [47,48], indicating the importance of IDPs, not in the beginning of life, but in life’s blossoming. However, just as pluripotent proteins appear to have left a “fossil imprint” on further proteome evolution (Figure 2) [22], it might turn out that IDPs were important in aspects of early biomolecular evolution. For example, one could envision a number of IDP precursors that could be induced into collapsing, therefore increasing their capacity for recruitment into folds. A further study on the capacity of random peptides with IDP-like composition to be involved in recruitment events could resolve this possibility.

### 4.5. Backbone Hydrogen Bonds Could Discretize Random Collapsed Structures into Proto-Folds

Despite the dynamic frustration displayed by random oily peptides, such peptides would still be capable of forming directed intermolecular interactions, due to the hydrogen bond donors and acceptors available at every backbone nitrogen and oxygen, respectively. It is possible that certain metastable collapsed forms available to oily pluripotent proteins could be further discretized into hierarchical proto-folds by the formation of secondary structure, which primarily requires backbone-backbone hydrogen bonding [49] and backbone-backbone proximity (collapsedness). Such hydrogen-bond-stabilized proto-folds could have served as evolutionary intermediates to the well-folding conformations that we find today. It is not known whether the allowance to readily form discrete (backbone stabilized) secondary structures increases the utility of peptides as an evolvable and functional polymer. It is, however, interesting to find that analogs to peptides—peptoids, which lack the capacity to form backbone-backbone hydrogen bonds—were not co-opted by biological systems, despite their abundance in abiotic conditions (e.g., [50]).

### 4.6. Detailed Mechanisms

The utility of pluripotent peptides has been discussed in the most general case, so as to accommodate differences in detailed mechanisms. For example, multiple short pluripotent peptides (inserts) could be strung together in multiple arrangements, thereby resulting in a rich set of possible outcomes and combinations of secondary structural elements. The mechanisms utilized in *ab initio* invention may even be similar to those utilized today for the diversification [33,34] of stable folds through “tinkering” [18] (such as recombination, deletions, insertions and point mutations) [13]; the thesis of this hypothesis is that pluripotent peptides would have increased the probability of *ab initio* fold invention regardless of the specific mechanism. It is for this reason that the interesting particulars of the *ab initio* invention process, such as the exact types of amino acids available during biogenesis [51] and the evolution of the particular triplet codon system [52,53], are left uncoupled to the pluripotent process; the hypothesis only depends on the presence of oily amino acids within the prebiotic repertoire (which is indicated in [51]).

### 4.7. Importance of Charged and Polar Amino Acids

While oily amino acids may be important in fold evolvability, they do not display substantial catalytic activity. For this reason, a completely oily peptide is not likely to be useful in a (pre)evolutionary setting. However, the presence of some polar and charged amino acids (already known to be important for cofactor binding and catalytic activity in today’s proteins [1]) could result in the successful recruitment of these “augmented” oily pluripotent peptides. Such recruiting events (discussed below) become even more plausible, given that polar amino acids describe limited catalytic activity even in free and peptide form [54], and, therefore, would likely describe residual activity in pluripotent peptide settings.

### 4.8. Recruiting Events

The transition from random peptide to folded protein would have occurred through “recruiting events” (shown as downward arrows in Figure 1c), where single folds within a pluripotent peptide’s structural ensemble may be stabilized due to arbitrary recruiting or “pull down” events, caused by, for example, chance interactions with other organic and inorganic molecules. Possibly, these pluripotent peptides, coupled with other stabilizing factors (e.g., early “co-factors”), were the initial canvases upon which natural selection was manifested. This concept is supported by the notion that only a few “nucleating” contacts are required to stabilize one fold over another [55,56,57]. Interestingly, in bioinformatics studies, all putatively “early” instances of new folds were found to be strongly coupled to the appearance of (or recruitment by) metals and other inorganic molecules [58,59], while later instances of such folds may shed their mandatory “dependence” on the cofactor [59].

## 5. Discussions Regarding Biogenesis

Note that events, such as recruiting events, have been intentionally stripped of specific chemical mechanisms, so as to allow for both protein-world and RNA-world hypotheses associated with metabolism-first [60,61,62,63,64] and replication-first [65,66,67] scenarios of the origins of life, respectively. This section entertains the possible utility of pluripotent peptides in biogenesis sans agency by RNA and should be considered as supplementary to the hypothesis.

### 5.1. Pre-Proteome: A Rich Protein Repertoire, Prelife

**Figure 4:**
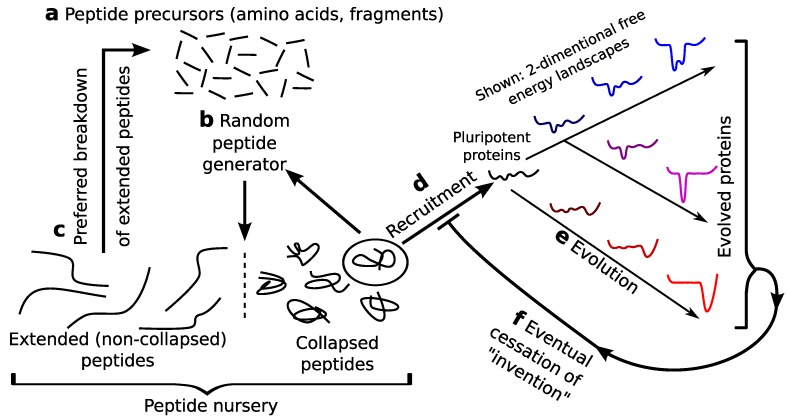
A cartoon representation of a prebiotic pluripotent peptide nursery. The prebiotic presence of amino acids (**a**) [8,9] and peptide generators (**b**) [10,11,12], along with the preferential degradation of extended *versus* collapsed peptides (**c**) [19,68] indicate that a dominantly oily and, hence, evolvable [21] or pluripotent species could have populated a prebiotic setting. This raises the potential for the recruitment of pluripotent peptide in prebiotic or neobiotic settings (**d**).

This section focuses on a scenario (Figure 4) where oily (pluripotent) peptides could be enriched in prebiotic pools that could then serve as nurseries for pre-biological recruitment. This scenario is particularly plausible given the prebiotic presence of amino acids [4,5,7,8,9] (Figure 4a) and inorganic peptide generators [10,11,12] (Figure 4b), along with the increased susceptibility to chemical degradation for extended (non-oily) peptides (hastening the step in Figure 4c) [19,68]. Such self-perpetuating protein soups could have been producing a rich, but randomly varying, repertoire of semi-stable protein folds and functions well before the beginning of life.

### 5.2. Potentially the First Recruiting Event

Assuming an environment where a peptide generator exists [10,11,12] and peptide degradation happens [19,68], a pluripotent peptide nursery could be perpetuated (Figure 4). This perpetuation would persist, initially due to the presence of the original (inorganic) peptide generators. However, with the presence of pluripotent peptides in the mix, the first recruitment event could likely have been caused by the stabilization of a particular fold by the inorganic molecule that generates peptides themselves. This would be the first prebiotic enzyme that creates other peptides. Such transient pluripotent peptides with stabilized folds and functionalities, while not necessarily undergoing natural selection (and allele transference or inheritance), must undergo chemical “selection”, given the following facts: (i) more collapsed peptides will persist longer in reactive pools [19,68]; and (ii) complexed peptides (newly recruited enzymes) would be the most collapsed, due to the presence of additional favorable interactions. The idea that a pluripotent peptide could catalyze peptide formation is not unfounded, given the presence of non-ribosomal peptide synthetases [69,70,71,72].

### 5.3. An Open Call to Experimentalists

Evidence for the pluripotent hypothesis has been obtained mostly from broad observations of proteomes over evolutionary time [22], coarse-grained peptide model simulations [21], and relationships between dynamism and evolvability [23]. While well beyond the scope of this paper, it would be interesting to eventually obtain insights for, or against, the pluripotent hypothesis via synthetic biology. It is possible today to combinatorially generate random peptides that are selected to bind molecules, such as adenosine triphosphate (ATP) [73]. One similar study that would help resolve the utility of random oily peptides would be to maintain a soup where random peptides are both steadily generated and susceptible to chemical degradation, in the presence of numerous small molecules, such as tagged ATP or metal clusters. It would be interesting to track both the oil content of longer chain species (the expectation is that these would be more oily than random), as well as the co-localization (and presumed binding) of these chains to the tagged molecules, which would result in more collapsed and less susceptible complexes that would be experimental evidence for the first prebiotic recruiting event sans directed pressures.

### 5.4. Ending Remarks

This paper provides a simple evidence-supported conjecture: that most protein folds emerged from pluripotent peptides, which are typified in the random peptide world by oily peptides. The recruiting events that result in *ab initio* fold invention [13] would have occurred before the beginning/diversification of fully developed single-celled organisms. The scenario discussed (Figure 4) indicates that as long as oily amino acids exist within the repertoire of amino acids, each step involved in going from random peptide to oily, but biologically active, peptide (and, hence, protein) is expected and potentially spontaneous, albeit at possibly long timescales. Such a notion reflects Oparin’s hypothesis [61] that the origin of life may not be an improbable and fleeting spark, but a process that is inevitable in the right situation. I will leave the reader with Oparin’s thoughts on the matter, stating the same with greater clarity [61].

“Life precursors—high molecular compounds, probionts and primitive living entities—repeatedly developed, disintegrated and emerged again in different places and at various times. Therefore, primitive organisms must have co-existed for a long time (probably, for many hundred million years) with simpler representatives of earlier biopoetic stages that had developed in other ‘subvital territories’. This modern concept, assuming multiplicity of the emergence of living beings, rules out entirely the former hypothesis that postulated an accidental emergence of life—the rarest phenomenon which could have happened only once during the whole period of the existence of the Earth”

(Oparin, 1976, Origins of Life, vol. 7 p. 3)

## References

[1] Berg J.M., Tymoczko J.L., Stryer L. (2010). Biochemistry, International Edition.

[2] Cooper G.M., Hausman R.E. (2013). The Cell: A Molecular Approach.

[3] Chothia C., Hubbard T., Brenner S., Barns H., Murzin A. (1997). Protein folds in the all-beta and all-alpha classes. Annu. Rev. Biophys. Biomol. Struct..

[4] Anders E. (1989). Pre-biotic organic matter from comets and asteroids. Nature.

[5] Zahnle K., Grinspoon D. (1990). Comet dust as a source of amino acids at the Cretaceous/Tertiary boundary. Nature.

[6] Oró J., Miller S.L., Lazcano A. (1990). The origin and early evolution of life on Earth. Annu. Rev. Earth Planet. Sci..

[7] Glavin D., Dworkin J., Sandford S. (2008). Detection of cometary amines in samples returned by Stardust. Meteorit. Planet. Sci..

[8] Miller S.L. (1953). A production of amino acids under possible primitive earth conditions. Science.

[9] Matthews C.N., Moser R.E. (1967). Peptide synthesis from hydrogen cyanide and water. Nature.

[10] Plankensteiner K., Reiner H., Rode B.M. (2005). Catalytically increased prebiotic peptide formation: Ditryptophan, dilysine, and diserine. Orig. Life Evol. Biosph..

[11] Wachtershauser G. (2007). On the chemistry and evolution of the pioneer organism. Chem. Biodivers..

[12] Leman L., Orgel L., Ghadiri M.R. (2004). Carbonyl sulfide-mediated prebiotic formation of peptides. Science.

[13] Chothia C., Gough J., Vogel C., Teichmann S. (2003). Evolution of the Protein Repertoire. Science.

[14] Blanck A., Oesterhelt D., Ferrando E., Schegk E.S., Lottspeich F. (1989). Primary structure of sensory rhodopsin I, a prokaryotic photoreceptor. EMBO J..

[15] Gough J. (2005). Convergent evolution of domain architectures (is rare). Bioinformatics.

[16] Koonin E.V., Aravind L., Kondrashov A.S. (2000). The impact of comparative genomics on our understanding of evolution. Cell.

[17] Kurland C.G., Canback B., Berg O.G. (2003). Horizontal gene transfer: A critical view. Proc. Natl. Acad. Sci. USA.

[18] Jacob F. (1977). Evolution and tinkering. Science.

[19] Abkevich V.I., Gutin A.M., Shakhnovich E.I. (1996). How the first biopolymers could have evolved. Proc. Natl. Acad. Sci. USA.

[20] Caetano-Anollés G., Wang M., Caetano-Anollés D., Mittenthal J.E. (2009). The origin, evolution and structure of the protein world. Biochem. J..

[21] Mannige R.V. (2013). Two regimes of protein evolution and their unique dependencies on sequence composition. Phys. Rev. E.

[22] Mannige R.V., Brooks C.L., Shakhnovich E.I. (2012). A universal trend among proteomes indicates an oily last common ancestor. PLoS Comput. Biol..

[23] Mannige R. (2014). Dynamic new world: Refining our view of protein structure, function and evolution. Proteomes.

[24] James L.C., Roversi P., Tawfik D.S. (2003). Antibody multispecificity mediated by conformational diversity. Science.

[25] James L.C., Tawfik D.S. (2003). Conformational diversity and protein evolution–a 60-year-old hypothesis revisited. Trends Biochem. Sci..

[26] Tokuriki N., Tawfik D.S. (2009). Protein dynamism and evolvability. Science.

[27] Soskine M., Tawfik D.S. (2010). Mutational effects and the evolution of new protein functions. Nat. Genet..

[28] Yadid I., Kirshenbaum N., Sharon M., Dym O., Tawfik D.S. (2010). Metamorphic proteins mediate evolutionary transitions of structure. Proc. Natl. Acad. Sci. USA.

[29] Pauling L. (1940). A Theory of the Structure and Process of Formation of Antibodies. J. Am. Chem. Soc..

[30] Landsteiner K. (1936). The Specificity of Serological Reactions.

[31] Zimmermann J., Oakman E.L., Thorpe I.F., Shi X., Abbyad P., Brooks C.L., Boxer S.G., Romesberg F.E. (2006). Antibody evolution constrains conformational heterogeneity by tailoring protein dynamics. Proc. Natl. Acad. Sci. USA.

[32] Thorpe I.F., Brooks C.L. (2007). Molecular evolution of affinity and flexibility in the immune system. Proc. Natl. Acad. Sci. USA.

[33] Jensen R.A. (1976). Enzyme recruitment in evolution of new function. Annu. Rev. Microbiol..

[34] Jensen R.A., Gu W. (1996). Evolutionary recruitment of biochemically specialized subdivisions of Family I within the protein superfamily of aminotransferases. J. Bacteriol..

[35] Bentley S.D., Parkhill J. (2004). Comparative genomic structure of prokaryotes. Annu. Rev. Genet..

[36] Jordan I.K., Kondrashov F.A., Adzhubei I.A., Wolf Y.I., Koonin E.V., Kondrashov A.S., Sunyaev S. (2005). A universal trend of amino acid gain and loss in protein evolution. Nature.

[37] Zeldovich K.B., Berezovsky I.N., Shakhnovich E.I. (2007). Protein and DNA sequence determinants of thermophilic adaptation. PLoS Comput. Biol..

[B38-proteomes-02-00154] 38.Note that affinity maturation is only used as an example to indicate the importance of a transition from a dynamic to rigid state; the actual *mode* of maturation is far too specialized to be generalized as a relevant mechanism of evolution.

[39] Bryngelson J.D., Wolynes P.G. (1987). Spin glasses and the statistical mechanics of protein folding. Proc. Natl. Acad. Sci. USA.

[40] Onuchic J.N., Luthey-Schulten Z., Wolynes P.G. (1997). Theory of protein folding: The energy landscape perspective. Annu. Rev. Phys. Chem..

[41] Chiti F., Dobson C.M. (2006). Protein misfolding, functional amyloid, and human disease. Annu. Rev. Biochem..

[42] Ellis R.J. (2001). Macromolecular crowding: Obvious but underappreciated. Trends Biochem. Sci..

[43] Hanczyc M.M., Toyota T., Ikegami T., Packard N., Sugawara T. (2007). Fatty acid chemistry at the oil-water interface: Self-propelled oil droplets. J. Am. Chem. Soc..

[44] Toyota T., Maru N., Hanczyc M.M., Ikegami T., Sugawara T. (2009). Self-propelled oil droplets consuming “fuel” surfactant. J. Am. Chem. Soc..

[45] Horibe N., Hanczyc M., Ikegami T. (2011). Mode Switching and Collective Behavior in Chemical Oil Droplets. Entropy.

[46] Dunker A.K., Babu M.M., Barbar E., Blackledge M., Bondos S.E., Dosztányi Z., Dyson H.J., Forman-Kay J., Fuxreiter M., Gsponer J. (2013). What’s in a name? Why these proteins are intrinsically disordered?. Intrinsically Disord. Proteins.

[47] Oldfield C.J., Cheng Y., Cortese M.S., Brown C.J., Uversky V.N., Dunker A.K. (2005). Comparing and combining predictors of mostly disordered proteins. Biochemistry.

[48] Schad E., Tompa P., Hegyi H. (2011). The relationship between proteome size, structural disorder and organism complexity. Genome Biol..

[49] Pauling L., Corey R.B., Branson H.R. (1951). The structure of proteins: Two hydrogen-bonded helical configurations of the polypeptide chain. Proc. Natl. Acad. Sci. USA.

[50] Pizzarello S., Schrader D.L., Monroe A.A., Lauretta D.S. (2012). Large enantiomeric excesses in primitive meteorites and the diverse effects of water in cosmochemical evolution. Proc. Natl. Acad. Sci. USA.

[51] Longo L.M., Lee J., Blaber M. (2013). Simplified protein design biased for prebiotic amino acids yields a foldable, halophilic protein. Proc. Natl. Acad. Sci. USA.

[52] Trifonov E.N. (2000). Consensus temporal order of amino acids and evolution of the triplet code. Gene.

[53] Trifonov E.N. (2004). The triplet code from first principles. J. Biomol. Struct. Dyn..

[54] Bar-Nun A., Kochavi E., Bar-Nun S. (1994). Assemblies of Free Amino Acids as Possible Prebiotic Catalysts. J. Mol. Evol..

[55] Mirny L.A., Abkevich V.I., Shakhnovich E.I. (1998). How evolution makes proteins fold quickly. Proc. Natl. Acad. Sci. USA.

[56] Mirny L.A., Shakhnovich E.I. (1999). Universally conserved positions in protein folds: Reading evolutionary signals about stability, folding kinetics and function. J. Mol. Biol..

[57] Mirny L., Shakhnovich E. (2001). Evolutionary conservation of the folding nucleus. J. Mol. Biol..

[58] Ji H.F., Kong D.X., Shen L., Chen L.L., Ma B.G., Zhang H.Y. (2007). Distribution patterns of small-molecule ligands in the protein universe and implications for origin of life and drug discovery. Genome Biol..

[59] Ji H.F., Chen L., Jiang Y.Y., Zhang H.Y. (2009). Evolutionary formation of new protein folds is linked to metallic cofactor recruitment. Bioessays.

[60] Oparin A.I., Bernal J.D. (1967). Proiskhozhdenie zhizny [Origin of life], Izd.

[61] Oparin A.I. (1976). Evolution of the concepts of the origin of life, 1924–1974. Orig. Life.

[62] Cairns-Smith A.G. (1966). The origin of life and the nature of the primitive gene. J. Theor. Biol..

[63] King G.A. (1977). Symbiosis and the origin of life. Orig. Life.

[64] Kauffman S.A. (1986). Autocatalytic sets of proteins. J. Theor. Biol..

[65] Eigen M. (1971). Molecular self-organization and the early stages of evolution. Experientia.

[66] Orgel L.E. (1994). The origin of life on the earth. Sci. Am..

[67] Lazcano A., Miller S.L. (1996). The origin and early evolution of life: Prebiotic chemistry, the pre-RNA world, and time. Cell.

[68] Abkevich V.I., Gutin A.M., Shakhnovich E.I. (1997). Computer simulations of prebiotic evolution. Pac. Symp. Biocomput..

[69] Marahiel M.A., Stachelhaus T., Mootz H.D. (1997). Modular Peptide Synthetases Involved in Nonribosomal Peptide Synthesis. Chem. Rev..

[70] Lautru S., Challis G.L. (2004). Substrate recognition by nonribosomal peptide synthetase multi-enzymes. Microbiology.

[71] Straight P.D., Fischbach M.A., Walsh C.T., Rudner D.Z., Kolter R. (2007). A singular enzymatic megacomplex from Bacillus subtilis. Proc. Natl. Acad. Sci. USA.

[72] Strieker M., Tanović A., Marahiel M.A. (2010). Nonribosomal peptide synthetases: Structures and dynamics. Curr. Opin. Struct. Biol..

[73] Keefe A.D., Szostak J.W. (2001). Functional proteins from a random-sequence library. Nature.

